# Artificial Light at Night Reduces Anxiety-like Behavior in Female Mice with Exacerbated Mammary Tumor Growth

**DOI:** 10.3390/cancers13194860

**Published:** 2021-09-28

**Authors:** William H. Walker, Raegan M. Kvadas, Laura E. May, Jennifer A. Liu, Jacob R. Bumgarner, James C. Walton, A. Courtney DeVries, Robert T. Dauchy, David E. Blask, Randy J. Nelson

**Affiliations:** 1 Department of Neuroscience, Rockefeller Neuroscience Institute, West Virginia University, Morgantown, WV 26505, USA; rk0035@mix.wvu.edu (R.M.K.); lem0027@mix.wvu.edu (L.E.M.); jal0075@mix.wvu.edu (J.A.L.); jrbumgarner@mix.wvu.edu (J.R.B.); james.walton@hsc.wvu.edu (J.C.W.); courtney.devries@hsc.wvu.edu (A.C.D.); randy.nelson@hsc.wvu.edu (R.J.N.); 2 Department of Medicine, Division of Oncology/Hematology, West Virginia University, Morgantown, WV 26505, USA; 3 Cancer Institute, West Virginia University, Morgantown, WV 26505, USA; 4 Department of Structural and Cellular Biology, Tulane University School of Medicine, New Orleans, LA 70112, USA; rdauchy@tulane.edu (R.T.D.); dblask@tulane.edu (D.E.B.)

**Keywords:** circadian rhythms, breast cancer, dim light at night, tumor growth, circadian disruption

## Abstract

**Simple Summary:**

Artificial light at night, initially assumed to be innocuous, is associated with an increased risk for developing mood disorders, sleep disturbances, and cancer. However, the influence of ALAN on affective behavior in tumor-bearing mice has not been investigated. Here, we demonstrate that ALAN reduces the latency to tumor onset and increases terminal tumor volume. Additionally, tumor-bearing mice housed in dark nights exhibit increased anxiety-like behavior which is prevented via housing in ALAN.

**Abstract:**

Artificial light at night (ALAN) is a pervasive phenomenon. Although initially assumed to be innocuous, recent research has demonstrated its deleterious effects on physiology and behavior. Exposure to ALAN is associated with disruptions to sleep/wake cycles, development of mood disorders, metabolic disorders, and cancer. However, the influence of ALAN on affective behavior in tumor-bearing mice has not been investigated. We hypothesize that exposure to ALAN accelerates mammary tumor growth and predict that ALAN exacerbates negative affective behaviors in tumor-bearing mice. Adult (>8 weeks) female C3H mice received a unilateral orthotropic injection of FM3A mouse mammary carcinoma cells (1.0 × 10^5^ in 100 μL) into the fourth inguinal mammary gland. Nineteen days after tumor inoculation, mice were tested for sucrose preference (anhedonia-like behavior). The following day, mice were subjected to an open field test (anxiety-like behavior), followed by forced swim testing (depressive-like behavior). Regardless of tumor status, mice housed in ALAN increased body mass through the first ten days. Tumor-bearing ALAN-housed mice demonstrated reduced latency to tumor onset (day 5) and increased terminal tumor volume (day 21). Exposure to ALAN reduced sucrose preference independent of tumor status. Additionally, tumor-bearing mice housed in dark nights demonstrated significantly increased anxiety-like behavior that was normalized via housing in ALAN. Together, these data reaffirm the negative effects of ALAN on tumorigenesis and demonstrate the potential anxiolytic effect of ALAN in the presence of mammary tumors.

## 1. Introduction

The suprachiasmatic nucleus (SCN) of the hypothalamus functions as the master circadian clock in mammals. This nucleus of approximately 50,000 neurons in humans and ~10,000–20,000 neurons in rodents is responsible for maintaining proper synchronization of behavioral and biological processes with the external 24-h day [1]. This synchronization occurs via entrainment of the circadian system to multiple environmental stimuli such as light/dark cycles, availability of food, social interaction, and, to a lesser extent in mammals, environmental temperature [2,3,4,5]. However, light is the primary zeitgeber (“time giver”) that entrains the SCN [6,7,8]. Since the advent of electrical lighting approximately 140 years ago, this process has become increasingly disrupted.

It is estimated that approximately 99% of the population in the United States and Europe are currently exposed to artificial light at night (ALAN) [9]. Although initially thought to be innocuous, ALAN is associated with an increased risk for developing mood disorders, sleep disturbances, and cancer [10,11,12,13,14]. Indeed, rodent studies demonstrate the ability of LAN to increase tumorigenesis [15,16,17,18,19]. Exposure to as little as 0.2 lux or 30 min (3.10 × 10^20^ photon cm^−2^s^−1^) of ALAN is sufficient to accelerate mammary tumor growth [19,20]. This is hypothesized to occur via melatonin suppression by ALAN, as exogenous melatonin administration ameliorates ALAN effects on tumorigenesis [17,18,19,20,21,22].

The detrimental effects of ALAN are not specific to cancer. Studies in rodents demonstrate that dim ALAN leads to several detrimental effects, including increased body mass, blunted clock gene rhythms, decreased hippocampal neurotrophins, increased depressive-like behavior, and the development of neuroinflammation [21,22,23,24]. Recently, inflammation has been implicated as a possible mediator for the development of comorbid mood disorders in breast cancer survivors [25,26,27]. Indeed, numerous studies have demonstrated the ability of peripheral tumors to induce neuroinflammation [28,29,30,31,32]. Notably, comorbid mood disorders are associated with increased cancer progression, decreased quality of life, and reduced survival rates in cancer survivors [33,34,35,36].

Due to (1) the demonstrated relationship between ALAN and cancer [15,16,17,18,19], (2) the ability of ALAN to initiate neuroinflammation [21,22,23,24], and (3) the exaggerated inflammatory response to an immune challenge following exposure to ALAN [37], we sought to determine the effects of ALAN exposure on mammary tumorigenesis and affective behavior in C3H mice. We hypothesized that the exposure to dim light at night accelerates mammary tumor growth and exacerbates negative affective behaviors.

## 2. Methods

### 2.1. Animals and Experimental Outline

Sixty adult female (8 weeks old at the time of arrival) C3H mice were obtained from Charles River Laboratories (Wilmington, MA, USA). Mice were individually housed and allowed ad libitum access to reverse osmosis purified water and food (Envigo Teklad #2018). Mice were allowed one week to acclimate to a standard 14:10 light–dark cycle prior to any experimental manipulation. After one week of acclimation, mice received a unilateral orthotopic 100 μL injection of FM3A mouse mammary carcinoma cells (1 × 10^5^ cells per injection) or vehicle (DMEM) into the fourth inguinal mammary gland. Injections occurred from ZT5-ZT7 (i.e., 5–7 h after lights on). Following tumor inoculation or vehicle injection, mice were placed in either an LD room (14 h of 150 lux: 10 h of 0 lux; lights on 0400 and lights off 18:00) or a dim ALAN room (14 h of 150 lux: 10 h of 5 lux; lights on 0400 and lights off 18:00) corresponding to their randomly assigned groups. ALAN was supplied using LUMA5 LED light strips (Hitlights Inc., Chino, CA, USA; 1.5 W/ft, 5000 K “cool white”, 1200 lumens). Cages were placed equidistant from the light strip and light levels were measured inside each cage, from the center, with the light meter (Mavolux 5032C illuminance meter; Nurnberg, Germany) facing upward to ensure ~5 lux of light exposure. Body mass and tumor measurements were obtained every five days. Latency to tumor onset was determined by palpability on day 5. As tumors became palpable, tumor volumes were obtained using sliding calipers. Tumor volume was calculated using the following formula: tumor volume = (length × width^2^)/2 [31,38,39]. Tumor growth rates were calculated via a linear regression as previously described [40]. On the night of day 16 following tumor inoculations, mice underwent sucrose preference habituation. This habituation was repeated on the nights of days 17 and 18; sucrose preference testing (to assess anhedonia-like behavior) occurred on the night of day 19. The next day, mice were tested in the open field (to assess total locomotor activity and anxiety-like behavior), followed by the forced swim test (to assess depressive-like behavior). On day 21, approximately 12 h after the conclusion of behavior testing, body mass was recorded, and the animals were euthanized. All experiments were performed in accordance with NIH Animal Welfare guidelines and were approved by the West Virginia University Institutional Animal Care and Use Committee. One mouse was excluded from all analyses, as it did not develop tumors throughout the study due to a failed injection.

### 2.2. Cell lines and Orthotopic Injections

Mouse mammary carcinoma FM3A cells were obtained from the Japanese Collection of Research Bioresources Cell Bank (Osaka, Japan). These cells are derived from a spontaneously arising mammary tumor in a C3H mouse that was initially maintained through the peritoneal cavity of C3H stock mice before being established as a cell line [41,42,43]. Typical progression of these cells has been described previously [41,42,43]. FM3A cells are metastatic and have been reported to result in lymph node and lung metastases [42,43]. Cells were maintained as previously described [31]. Prior to injections, cells were tested for mycoplasma using the PlasmoTest kit (InvivoGen, San Diego, CA, USA) and were found to be free of any mycoplasma. Cells were diluted in DMEM (Gibco, Waltham, MA, USA) to a concentration of 1 × 10^5^ cells per 100 μL for injections. Mice were briefly anesthetized using isoflurane, placed supine, and a small 1–2 mm midline incision between the fourth and ninth inguinal mammary glands was made. The incision was manipulated to allow for visualization and injection of the fourth inguinal mammary gland. Following the injection, the incision was closed using 3M Vetbond Tissue Adhesive (3M, Maplewood, MN, USA).

### 2.3. Behavioral Testing

Sucrose preference testing: Sucrose testing occurred during nights 16–18 (habituation) and night 19 (experimental test). Two 15 mL falcon tubes, both containing water (habituation)—or one containing water and the other containing 3% sucrose solution (experimental test)—with rubber stoppers and metal sippers were placed in the mouse’s home cage at the start of the dark phase. The tubes were removed 5 h later and were weighed to determine the amount of liquid consumption from each tube. The falcon tubes were randomly placed on either side of the cage and were alternated each night of sucrose habituation or testing to control for side bias. Percent preference for the sucrose solution was calculated for each mouse.

Open field testing: On day 20, the mice were placed in a 36 cm × 36 cm polypropylene open field arena for 10 min. Their horizontal and vertical movements were detected by two sets of infrared sensors mounted on the sides of the box (Open Field Photobeam Activity System, San Diego Instruments Inc., San Diego, CA, USA.). The boxes are contained in cabinets to attenuate any outside noise or light. Total locomotor activity, central tendency (within the first 5 min), and number of rears were calculated and analyzed for each mouse.

Forced swim test: Approximately 15 min after open field testing, mice were placed for 5 min in a 5000 mL beaker filled with ~3500 mL of water at a temperature of approximately 27 °C. Mice were videotaped and their behavior was later scored by an experimenter unaware of treatment assignments using The Observer XT 8.0 software (Noldus, Leesburg, VA, USA). Time spent floating, latency to float, and number of floating bouts were calculated and analyzed.

### 2.4. Statistics

**Figure 1:**
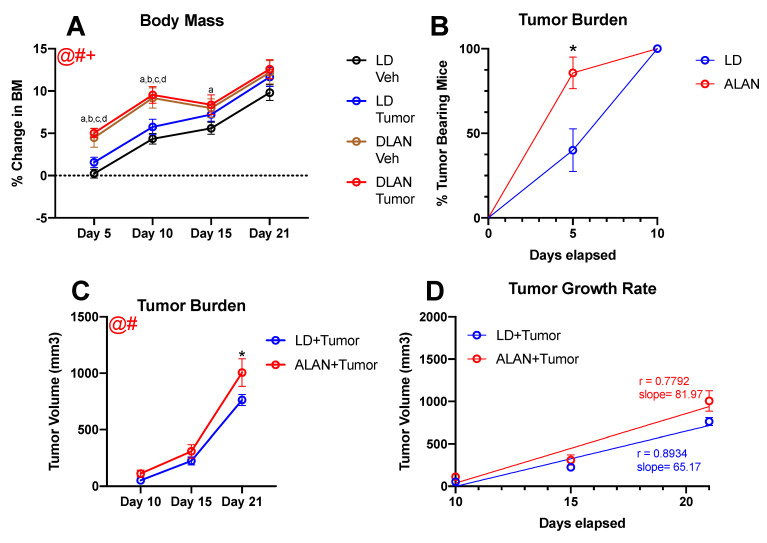
ALAN accelerates body mass gain and tumor burden: (**A**) ALAN accelerated body mass gain on days 5 and 10 regardless of injection type. Tumor-bearing mice housed in ALAN demonstrated reduced (**B**) latency to tumor onset and (**C**) increased tumor burden relative to LD-housed tumor-bearing mice. However, (**D**) tumor growth rate was not significantly different between groups. Error bars represent SEM; @ main effect of day, # main effect of lighting, + lighting by day interaction; (**A**) three-way repeated measures mixed-effect analysis (**B**) chi-squared test of independence (**C**) three-way repeated measures mixed-effect analysis; Fisher’s LSD multiple comparisons test. (a)—LD veh vs. ALAN tumor at *p* < 0.05. (b)—LD veh vs. ALAN veh at *p* < 0.05. (c)—LD tumor vs. ALAN veh at *p* < 0.05. (d)—LD tumor vs. ALAN tumor at *p* < 0.05. * LD tumor vs. ALAN tumor at *p* < 0.05. *n* = 13–15 per group.

**Figure 2:**
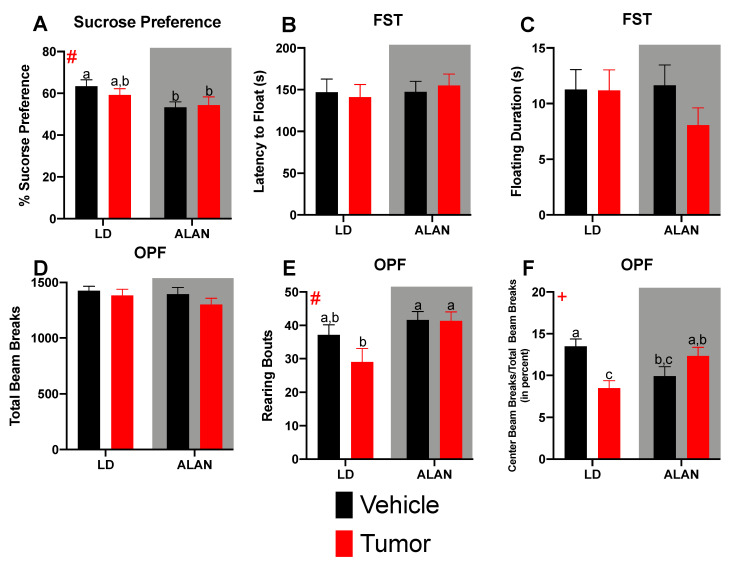
Mammary tumors and ALAN alter behavior: (**A**) ALAN significantly reduced sucrose preference. No effect was seen in (**B**) latency to float, (**C**) floating duration, or (**D**) total locomotor activity. (**E**) Tumor-bearing LD-housed mice displayed a significantly reduced number of rearing bouts relative to both ALAN-housed groups. (**F**) LD-housed tumor-bearing mice demonstrated significantly reduced central tendency relative to LD-housed vehicle-treated mice. This effect was normalized via housing in ALAN. Error bars represent SEM; # main effect of lighting, + lighting by day interaction; (**A**–**F**) two-way ANOVA; Fisher’s LSD multiple comparisons test. Graph bars that do not share a letter are statistically significantly different at *p* < 0.05. (a)—LD veh vs. ALAN tumor at *p* < 0.05. (b)—LD veh vs. ALAN veh at *p* < 0.05. (c)—LD tumor vs. ALAN veh at *p* < 0.05. *n* = 13–15 per group.

Outliers, defined as having a within-group Z score greater than 2, were removed a priori. No more than one outlier was removed from each experimental group. Body mass data (Figure 1A) were analyzed using a 3-way repeated measures mixed-effects analysis. To determine the effect of ALAN on latency to tumor onset, data (Figure 1B) were analyzed using a chi-square test of independence. Tumor burden (Figure 1C) was analyzed for mice bearing tumors using a 2-way repeated measures mixed-effects analysis. Tumor growth rates (Figure 1D) were analyzed using a linear regression that compared parallelism between lines (i.e., similar slope, then similar rate of tumor growth). Behavioral testing data (Figure 2A–F) were analyzed using a 2-way ANOVA. Following all ANOVAs, post hoc comparisons were made using Fisher’s LSD multiple comparison tests. Mean differences were considered statistically significant when *p* < 0.05. All statistical analyses were completed using GraphPad Version 9.0 (GraphPad Software Inc., San Diego, CA, USA).

## 3. Results

### 3.1. ALAN Accelerates Body Mass Gain

To determine whether the previously reported weight gain in response to ALAN was occurring in the present study, body mass measurements were obtained every five days beginning on day 5 [22]. There were significant main effects of lighting condition (F_1, 54_ = 15.15; *p* < 0.001), day (F_2, 361, 126.7_ = 111.9; *p* < 0.001), and a day by lighting interaction (F_3, 161_ = 4.158; *p* < 0.01) (Figure 1A). Regardless of tumor assignment, mice housed in ALAN demonstrated significantly increased percent change in body mass relative to mice in dark nights (LD) on days 5 and 10. Additionally, on day 15, tumor-bearing mice housed in ALAN displayed significantly increased weight gain relative to LD-housed vehicle-treated mice. No effect on body mass was seen on day 21.

### 3.2. ALAN Stimulates Tumor Growth

The effects of ALAN on latency to tumor onset were determined via the presence of palpable tumors on day 5. A higher percentage of mice housed in ALAN exhibited palpable tumors 5 days post-inoculation relative to a group of animals housed under completely dark nights (χ^2^ = 6.428; *p* < 0.05) (Figure 1B). Specifically, on day 5, 12 out of 14 ALAN mice exhibited palpable tumors compared to 6 out of 15 mice in LD conditions. Furthermore, ALAN exacerbated tumor burden (F_1, 27_ = 4.347; *p* < 0.05) (Figure 1C). Tumor-bearing mice housed in ALAN exhibited significantly increased tumor burden on day 21 relative to tumor-bearing mice housed in LD conditions. When comparing tumor growth rate, the slopes of the two lines were not significantly different between groups (F_1,81_ = 2.155; *p* = 0.14) (Figure 1D).

### 3.3. Mammary Tumors and ALAN Alter Behavior

To determine whether ALAN and mammary tumors resulted in additive determinantal effects on depressive-like and anxiety-like behavior, mice were tested for sucrose preference, forced swim testing, and open field behavior. There was a main effect of lighting on sucrose preference testing (F_1, 52_ = 5.443; *p* < 0.05) (Figure 2A). Regardless of whether tumor- or non-tumor-bearing, mice housed in ALAN demonstrated a significantly reduced sucrose preference relative to vehicle-treated LD-housed mice. Tumor-bearing LD-housed mice displayed an intermediate effect. No effects were seen in latency to float (Figure 2B), floating duration (Figure 2C), or the number of floating bouts (data not shown) during forced swim testing. Together, these data suggest a moderate depressive-like phenotype in ALAN-housed mice that is not worsened by the presence of a mammary tumor. Total locomotor activity was not significantly different among groups (Figure 2D). However, there was a significant effect of lighting on the number of rears during the open field test (F_1, 52_ = 6.996; *p* < 0.05) (Figure 2E). Tumor-bearing and vehicle-treated mice housed in ALAN displayed a significantly elevated number of rears relative to tumor-bearing LD-housed mice. Furthermore, there was a significant lighting by injection type interaction on central tendency (F_1, 53_ = 14.34; *p* < 0.001) (Figure 2F). LD-housed tumor-bearing mice demonstrated significantly increased anxiety-like behavior (i.e., reduced central tendency) relative to LD-housed vehicle-treated mice. Notably, housing mice in ALAN prevented the increased anxiety-like behavior in tumor-bearing mice.

## 4. Discussion

Worldwide, breast cancer is the most common cancer among women [44]. Breast cancer patients frequently experience detrimental symptoms, including alterations in mood, disrupted sleep/wake cycles, and cognitive deficits [45], all of which are associated with reduced quality of life and impaired survival [33,34,35,36,46,47,48]. ALAN is an equally pervasive phenomenon, with 99% of the population in the United States and Europe currently exposed to light at night (LAN) [9]. Similarly, ALAN has also been associated with alterations in mood, disrupted sleep/wake cycles, and cognitive deficits [12,23,24,49,50]. However, the potential interactive effects of ALAN and tumorigenesis on affective behavior had previously not been investigated. Therefore, the present study sought to examine the effects of ALAN exposure on mammary tumorigenesis and affective behavior in female C3H mice.

First, we examined the effects of ALAN on body mass. Regardless of tumor status, ALAN accelerated weight gain throughout the first ten days of the study (Figure 1A). Furthermore, tumor-bearing mice housed in ALAN maintained increased body mass gain relative to LD-housed vehicle-treated mice throughout the fifteen days of tumor development. Previous studies have reported increased body mass gain in response to ALAN [51,52,53,54]. Indeed, housing mice in 5 lux of ALAN for as little as 2 weeks increased body mass gain and altered whole-body metabolism [52]. The present study demonstrates that body mass changes can occur as soon as 5 days following housing in ALAN; these results suggest that it is likely ALAN is also altering whole-body metabolism in a shorter time frame than previously reported. Thus, future studies should examine the effect of acute ALAN exposure on whole-body metabolism in mice [55].

To determine the effects of ALAN on latency to tumor onset, mice were examined for the presence of palpable tumors on day 5. Tumor-bearing mice housed in ALAN demonstrated a significantly increased percentage of palpable tumors on day 5 relative to their LD-housed counterparts (Figure 1B). Indeed, 85% of ALAN-housed mice exhibited palpable tumors on day 5 vs. 40% of LD-housed mice. Similar effects were seen in tumor burden; ALAN-housed mice exhibited larger tumors throughout development and displayed significantly larger terminal tumor volume (Figure 1C). However, growth rates between groups were not statistically significantly different (Figure 1D). These data add to the growing literature detailing the relationship between ALAN and tumorigenesis. Epidemiological studies demonstrate the association of ALAN with breast cancer incidence [56,57,58,59]. Additionally, numerous foundational science studies have supported an association between ALAN exposure and breast cancer development [15,16,17,18,19]. Exposing nude rats to as little as 0.2 lux of LAN is sufficient to accelerate ERα + MCF-7 breast cancer xenograft growth [19,20,22]. Likewise, one 30 min period of ALAN (134 µ Wcm^−2^, 460 nm) was shown to increase tumor burden in 4T1 tumor-bearing BALB/c mice [19]. These effects are thought to occur via ALAN’s ability to suppress melatonin, as exogenous melatonin administration ameliorates ALAN effects on tumorigenesis [17,18,19,20]. In addition, disruption of melatonin rhythms via exposure to ALAN can lead to an increased number of metastases. Indeed, housing nude mice in ALAN led to increased metastatic developments in the lung, liver, and brain [60]. Administration of exogenous melatonin reduced tumor burden and the number of metastatic lesions, which, while not explicitly tested, appeared to suggest that the increased growth seen in the current study was likely due to suppressed melatonin rhythms via exposure to ALAN. Notably, the long photoperiod, shorter melatonin duration (reduced overall melatonin exposure during dark) in the controls may have contributed to there being no significant difference in growth rates/slopes. Exposure of control mice to a LD, 12:12 photoperiod, “normal” duration melatonin signal might have manifested as a greater/significant difference in growth rates between LD and ALAN groups as has been observed in our previous published studies.

Lastly, we examined the effects of ALAN exposure and mammary tumorigenesis on affective behavior. Regardless of tumor status, housing mice in ALAN reduced sucrose preference (Figure 2A), suggesting anhedonia. However, no effect was seen during forced swim testing (Figure 2B,C). Together, this suggests a moderate depressive-like phenotype in ALAN-housed animals. Notably, the presence of a mammary tumor had no effect on depressive-like behavior. These data are consistent with the previously reported increase in depressive-like behavior following exposure to ALAN [21,24,61]. Furthermore, previous studies from our lab have demonstrated no effects on depressive-like behavior following tumor inoculation via multiple murine mammary cell lines [31]. Of note, mice in the current study demonstrated high levels of activity during forced swim testing, which may have masked the previously reported effect of ALAN on this task. Thus, future studies utilizing the current strain of mice may want to consider other measurements of depressive-like behavior. Neither ALAN nor the presence of mammary tumors altered total locomotor activity in a novel environment (Figure 2D). However, LD-housed tumor-bearing mice exhibited increased anxiety-like behavior that was normalized in tumor-bearing mice exposed to ALAN. The increased anxiety-like behavior demonstrated in LD-housed tumor-bearing mice is consistent with foundational science and clinical data reporting increased anxiety in breast cancer survivors [62,63,64]. Notably, in foundational research, examination of anxiety-like behaviors in response to peripheral tumors is scarce outside of breast cancer. Thus, future studies should expand and examine the effects of other cancer types, as clinical anxiety symptoms are not specific to breast cancer [65,66]. The effect of ALAN exposure on anxiety-like behavior has produced varying results, as some studies report increased anxiety-like behavior following ALAN, whereas others report an anxiolytic effect [24,67]. This could be explained by differences in strains of mice, sex of mice, and length of ALAN exposure. In the current study, ALAN alone or the presence of a mammary tumor alone reduced central tendency relative to LD-housed mice. However, ALAN exposure plus a mammary tumor normalized anxiety-like behavior. To our knowledge, this is the first study to demonstrate a protective effect of ALAN in the presence of a tumor. Future studies should investigate the potential mechanism of this effect and determine the generalizability to other cancer types.

As with all experiments, there are limitations within the current study that must be addressed. First, in contrast to the animal models used in the present study, patients are aware of their cancer diagnosis. This awareness may bring with it fears of mortality which, in turn, are highly likely to contribute to depression and anxiety symptoms. Studying behavior in animal models allows for examination of affective behavior in tumor-bearing animals without the confound of fears of illness or mortality; this approach allows for the attribution of changes in affective behavior to tumors alone. The second limitation involved mice being housed on a 14:10 LD cycle, which may have reduced the difference in tumor growth between ALAN and LD-housed mice due to a shortened duration of melatonin secretion (i.e., 10 h). The mice were housed on a 14:10 LD cycle due to the potential confound of photoperiodic effects. Studies have demonstrated non-reproductive photoperiodic effects in *Mus musculus* [68,69]. Specifically relevant to tumor growth, studies have reported photoperiodic effects on immune function in *Mus musculus* [69]. LD 12:12 does not provide a distinct photoperiodic signal; thus, some mice may display short day phenotypes, whereas other mice may exhibit long day phenotypes depending on their individual response to the ambiguous day lengths. Similarly, a shortened temporal window between tumor measurements may have allowed for detection of more subtle differences in tumor growth rates between LD and ALAN-housed mice. However, increased tumor measurements would likely increase stress, which has demonstrated detrimental effects on both study outcomes [70,71]. Finally, the current study examines the effects of ALAN on affective behavior in tumor-bearing mice in one tumor model. Future studies should investigate generalizability to other cancer types.

In sum, the current study demonstrates a relationship among ALAN, tumorigenesis, and affective behavior. Exposure to ALAN yielded a transient increase in body mass gain. Additionally, ALAN accelerated the latency to tumor onset and increased tumor burden. Anhedonia was demonstrated in ALAN-housed mice. Further, tumor-bearing mice housed in dark nights had greater anxiety-like behavior that was altered by housing in ALAN. Together, these data reaffirm the negative effects of ALAN on tumorigenesis and demonstrate the potential anxiolytic effect of ALAN in the presence of mammary tumors.

## Data Availability

The data that support the findings of this study are available (in raw form) from the corresponding author upon reasonable request.
